# Micrometre resolution of a charge integrating microstrip detector with single photon sensitivity

**DOI:** 10.1107/S090904951200235X

**Published:** 2012-03-15

**Authors:** A. Schubert, A. Bergamaschi, C. David, R. Dinapoli, S. Elbracht-Leong, S. Gorelick, H. Graafsma, B. Henrich, I. Johnson, M. Lohmann, A. Mozzanica, V. Radicci, R. Rassool, L. Schädler, B. Schmitt, X. Shi, B. Sobott

**Affiliations:** a School of Physics, The University of Melbourne, Melbourne, Victoria 3010, Australia; b Paul Scherrer Institut, CH-5232 Villigen, Switzerland; c Deutsches Elektronen Synchrotron, DE-22607 Hamburg, Germany

**Keywords:** synchrotron radiation instrumentation, charge integrating, strip detectors

## Abstract

A synchrotron beam has been used to test the spatial resolution of a single-photon-resolving integrating readout-chip coupled to a 320 µm-thick silicon strip sensor with a dedicated readout system. Charge interpolation methods have yielded a spatial resolution of σ_*x*_ ≃ 1.8 µm for a 20 µm-pitch strip.

## Abstract 2

The spatial resolution of a single-photon-resolving integrating system has been improved using a non-linear charge interpolation approach. The resolution limit for a 20 µm-pitch sensor is presented.

## 1. Introduction

The advent of X-ray free-electron lasers (XFELs) such as the European XFEL brings new challenges in detector design. With photon fluences of the order of thousands or more per detector channel per bunch (bunch length ≃100 fs), single-photon-counting detectors are no longer feasible. The Paul Scherrer Institut (PSI) in collaboration with Deutsches Elektronen Synchrotron (DESY) has developed a single-photon-resolving integrating readout chip (GOTTHARD, gain optimizing microstrip system with analog readout) to cope with the high photon rates that will be produced at XFEL (Mozzanica *et al.*, 2010 ▶). A charge integrating readout can also be beneficial in synchrotron applications. Photon-counting detectors are rendered ineffective if charge is always shared between multiple strips (Bergamaschi *et al.*, 2008 ▶) as is the case for small strip pitches, necessitating the implementation of a charge integrating approach. Additionally, by utilizing analog information the spatial resolution of the system may be improved further *via* charge interpolation methods (Hubbeling *et al.*, 1991 ▶; Brenner *et al.*, 1993 ▶; Bergamaschi *et al.*, 2011 ▶).

The prototype of the charge integrating system, GOTTHARD, is briefly described in §2. The charge interpolation algorithm is outlined in §3, simulations in §4, and finally the experimental procedure and measured spatial resolution are presented in §5.

## 2. System description

**Figure 1:**
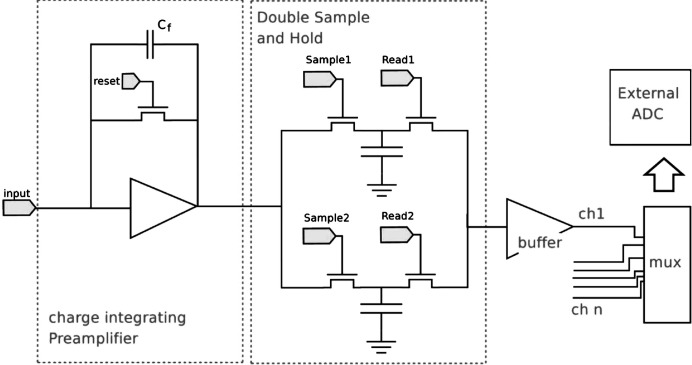
A simplified block diagram of the GOTTHARD chip.

The GOTTHARD prototype has been designed and integrated with a data acquisition (DAQ) system. The dynamic range and gain switching performance of GOTTHARD are detailed by Mozzanica *et al.* (2009 ▶). The chip is designed in UMC 0.25 µm technology and comprises 100 identical parallel channels. A simplified block diagram of a single channel is shown in Fig. 1 ▶. Each channel functions as a low-noise preamplifier with the small feedback capacitor providing the high gain necessary for single-photon resolution. Upon release of the reset switch, charge integration begins on the feedback capacitor such that the output voltage follows *V*_out_ = −*Q*_in_/*C*_f_. Dual sample and hold capacitors allow sampling of the output voltage pre- and post-integration time. The difference between the two readouts provides the integrated charge free from any reset noise contribution; this technique is termed correlated double sampling. At the end of each integration time the voltages are serially read out to an external analog-to-digital converter (ADC). To perform the high-resolution measurement, four chips are wire bonded to a 320 µm-thick multi-pitch silicon strip sensor designed by PSI and manufactured by Hamamatsu. The sensor contains pitches ranging from 10 to 25 µm in 5 µm increments, with multiple p+ implant and metalization configurations for each pitch. The DAQ system is based on a field-programmable gate array (FPGA). Analog readout is performed by two 14-bit 80 MHz ADCs. The digital outputs are buffered in the FPGA memory and transferred to an embedded processor, which is controlled by the user PC *via* a TCP/IP socket interface over 100 Mbit s^−1^ ethernet. This configuration allows system readout at frame rates up to 300 Hz.

## 3. Charge interpolation

High-energy physics has shown that by applying non-linear charge interpolation methods it is possible to improve the spatial resolution (Turchetta, 1993 ▶; Johnson *et al.*, 2004 ▶; Straulino *et al.*, 2006 ▶). It is possible to apply a similar principle to an X-ray detector with low noise as was shown by Mozzanica *et al.* (2010 ▶), where a simple analytical approach was used to achieve a spatial resolution of ∼3.3 µm r.m.s for a 20 µm pitch. Here, a non-linear interpolation approach, the η algorithm (Turchetta, 1993 ▶), is used to optimize the spatial resolution.

### 3.1. Charge sharing

Incident photons are converted to charge clouds within the sensor and are transported to collection electrodes by the applied electric field. Diffusion and electrostatic repulsion cause broadening of the charge clouds as they drift towards the collection electrode (Lutz, 1999 ▶). Charge sharing has been measured to occur in a region of 17 ± 3 µm between the strips, independent of the strip pitch, for a sensor with the same geometry and under equal biasing conditions as used here (Bergamaschi *et al.*, 2008 ▶). Therefore, small strip pitches will result in charge always being shared in two or more adjacent strips. As the strip pitch is increased, less charge sharing will occur as the area over which charge is fully collected by a strip increases. Consequently, for large pitches the charge interpolation is not effective over the central strip region and leads to degradation of the spatial resolution. In contrast, very small pitches have a higher inter-strip capacitance *C*_int_, resulting in increased noise, and charge may be shared on more than two strips, degrading the signal-to-noise ratio (SNR) which is defined as the ratio of the mean to the standard deviation of the pulse heights of single photons.

### 3.2. The η algorithm

**Figure 2:**
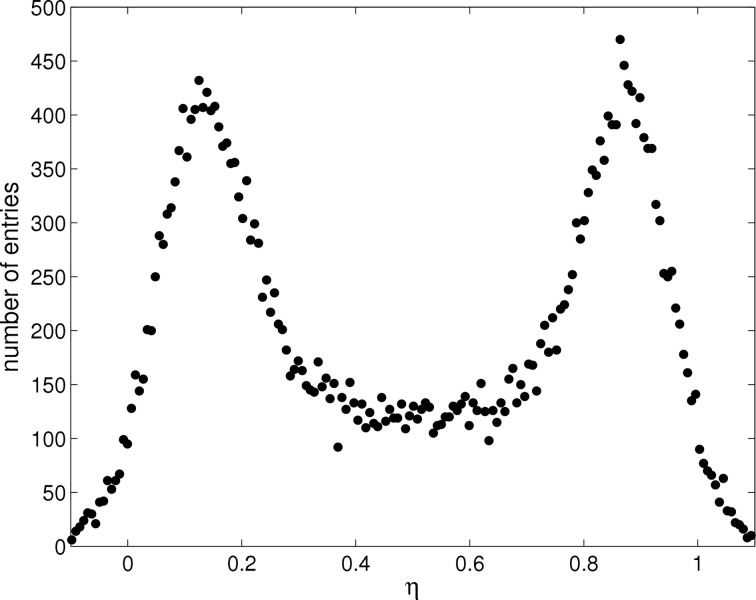
Experimentally obtained η distribution for two adjacent 20 µm-pitch strips in response to a 15 keV flat-field illumination. Lateral peaks are not centred at 0 and 1 owing to charge lost *via* capacitive coupling and charge sharing with neighbouring strips.

The variable η forms the basis of the charge interpolation scheme. If an isolated photon hit is considered, then η is defined as 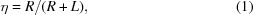
where *R* and *L* are the signals of the right and left channels in the pair, respectively. η may be considered as an average (weighted by the signals *L* and *R*) of the positions of the adjacent strips located at 0 and 1. The distribution in response to a flat-field illumination is shown in Fig. 2 ▶.

Since hits are uniformly distributed over the detector, the position of a hit 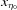 with respect to the left strip may be calculated from 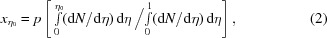
where d*N*/dη gives the differential η distribution and *p* is the strip pitch. Equation (2) defines a non-linear algorithm with d(η) given by the integral of the η distribution normalized to the total number of events in the distribution. The positions of the lateral peaks in the distribution shown in Fig. 2 ▶ are indicative of the degree of coupling between channels. The width of the η distribution peaks is inversely proportional to the SNR (Turchetta, 1993 ▶), from which the standard deviation of the Gaussian noise distribution, or equivalent-noise charge (ENC), is calculated to be 334 ± 8 e^−^ and 370 ± 19 e^−^ for the 25 and 20 µm-pitch channels, respectively. The noise values are in agreement with previously published results using pulse height distribution analysis (Mozzanica *et al.*, 2009 ▶).

## 4. Simulation

For optimization of the reconstruction, algorithm simulations of 20 and 25 µm-pitch sensors are performed as these yielded good results in previous experiments.

*Geant4* (Agostinelli *et al.*, 2003 ▶), a toolkit for the simulation of particle interaction with matter which is widely used in high-energy and nuclear physics as well as medical applications, is used to generate the initial charge distribution caused by photons impinging on the sensor. Charge transport and charge collection are based on a TCAD (technology computer aided design) simulation (Schubert *et al.*, 2010 ▶) using finite-element-analysis methods to solve equations responsible for charge transport, generation and recombination. To achieve the required submicrometre spatial resolution over the width of multiple strips, a two-dimensional approach is implemented owing to computational limitations. The simulation is used to study the effects of strip pitch, implant width and sensor thickness and, as a result of this, interstrip capacitance and noise on the performance of the reconstruction algorithm.

**Figure 3:**
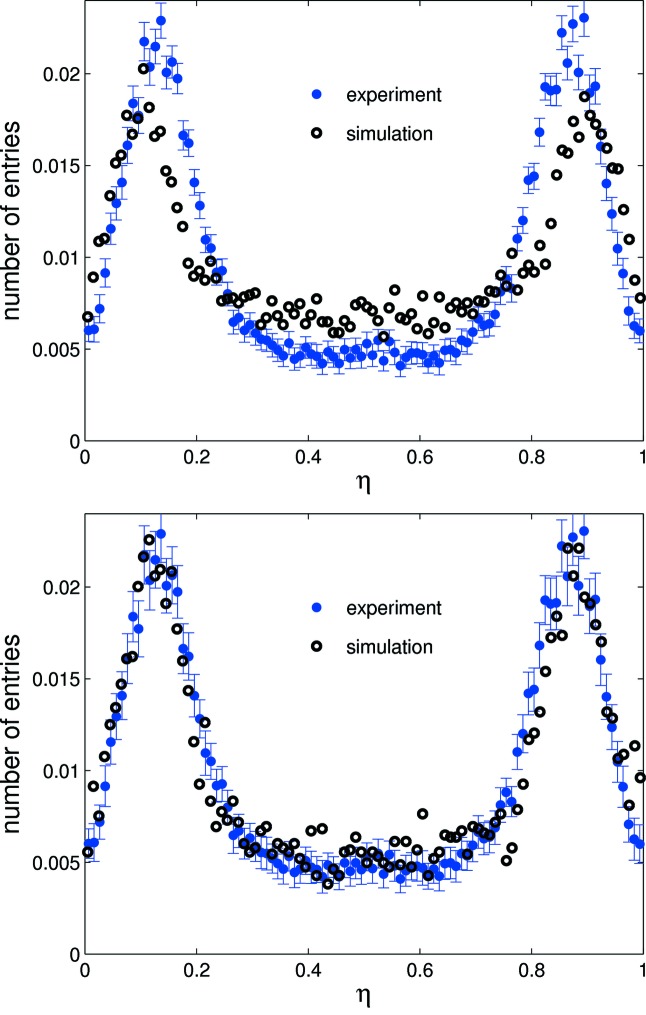
Top: experimental and simulated η distributions for a 25 µm-pitch strip sensor, normalized to integrated counts. The simulated peaks are closer to 0 and 1 than in the experimental distribution owing to reduced charge sharing as a result of underestimating the strip coupling in the simulation. Bottom: introduction of the coupling factor *K* = 0.06 yields agreement within experimental error between experiment and simulation.

A comparison of experimental and simulated η distributions in response to a flat-field illumination is shown in Fig. 3 ▶. The lateral peaks of the simulated distribution are nearer to 0 and 1 than those of its experimental counterpart, indicating the simulation underestimates coupling between strips. This is due to the omission of the preamp and more specifically the charge integration occurring on the feedback capacitor in the simulation. For the preamp only the noise is simulated by adding a random noise with a Gaussian distribution to the integrated charge. In the real detector the input of the preamp, and with this the strip, in the sensor has a certain voltage swing during charge integration owing to the limited DC gain of the preamp. This voltage modulation then couples a strip to its neighbours *via* interstrip capacitances causing a cross-talk between neighbouring strips. In the simulation this cross-talk is lacking and therefore needs to be compensated for *via* the introduction of a coupling factor, *K*.

*K* is defined as the proportion of charge shared with adjacent strips for the integrated charge per strip for each photon interaction. Simulation shows that a small increase in coupling (see Fig. 3 ▶) causes the lateral peaks in the η distribution to shift towards the centre owing to increased charge lost to neighbouring strips. *K* ranges from ∼0.05 to 0.07 depending on the strip geometry (pitch, implant size, metalization); this agrees well with the 7% coupling measured for GOTTHARD. The excellent agreement between simulation and experiment provides confidence in the simulation’s predictions, allowing it to be used to explain the origins of features in the experimental data.

To quantify the effectiveness of the reconstruction algorithm a reconstruction error Σ_R_ is defined for the simulation as Σ_R_ = *x*_*i*_ − μ_*i*_, where *x*_*i*_ is the reconstructed position for hit *i* and μ_*i*_ is the corresponding injection position.

**Figure 5:**
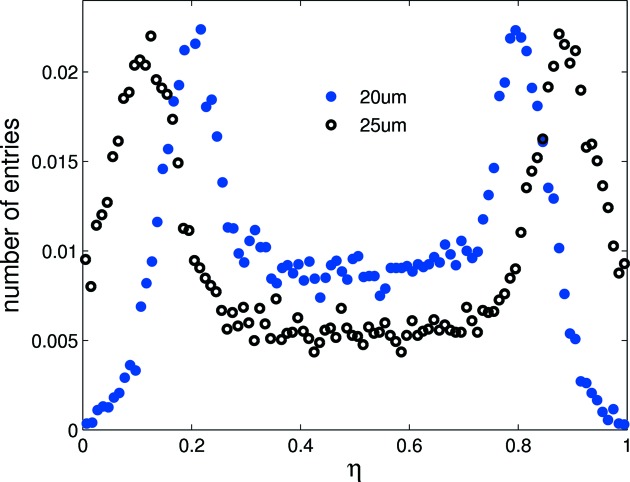
Simulated η distribution of the 20 and 25 µm pitches for a coupling of *K* = 0.06 and ENC = 390 e^−^ reconstructed from a flat-field distribution. Compared with the 25 µm pitch, the peaks of the 20 µm-pitch η distribution exhibit a significant shift towards the centre of the η distribution and a higher plateau at the centre, indicating a greater degree of charge sharing.

**Figure 6:**
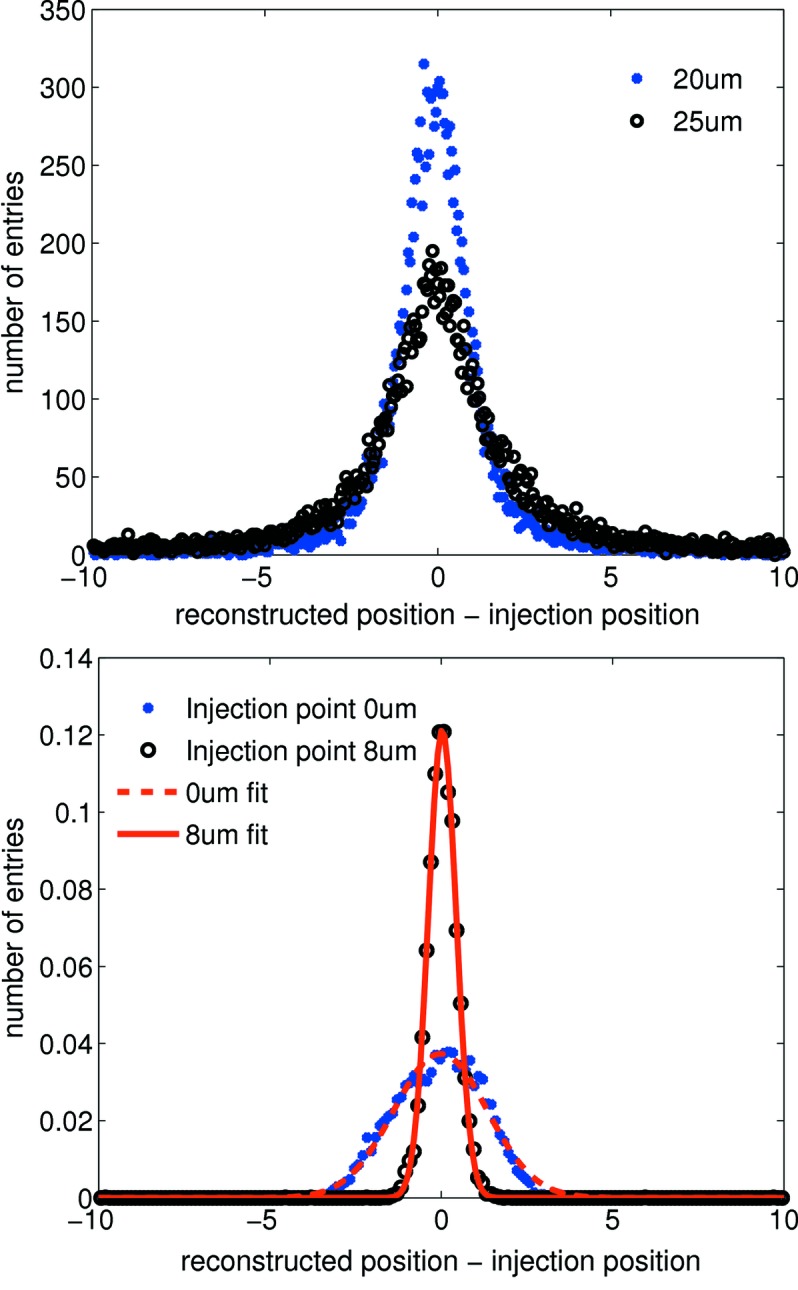
Top: reconstruction error (difference between reconstructed and injection position) for a simulated flat-field illumination of the 20 and 25 µm pitches for an ENC of 340 e^−^. The smaller pitch shows a smaller reconstruction error. Bottom: reconstruction error distributions for the 20 µm-pitch strip for injection positions 0 µm (centred on the left strip) and 8 µm (close to the strip border) shown for an ENC of 340 e^−^. The differences between the two distributions are due to little charge sharing at injection point 0 µm. Gaussian fits give a reconstruction error σ_*x*_ of 1.38 ± 0.04 µm at the strip centre and 0.40 ± 0.03 µm close to the strip boundary.

**Figure 7:**
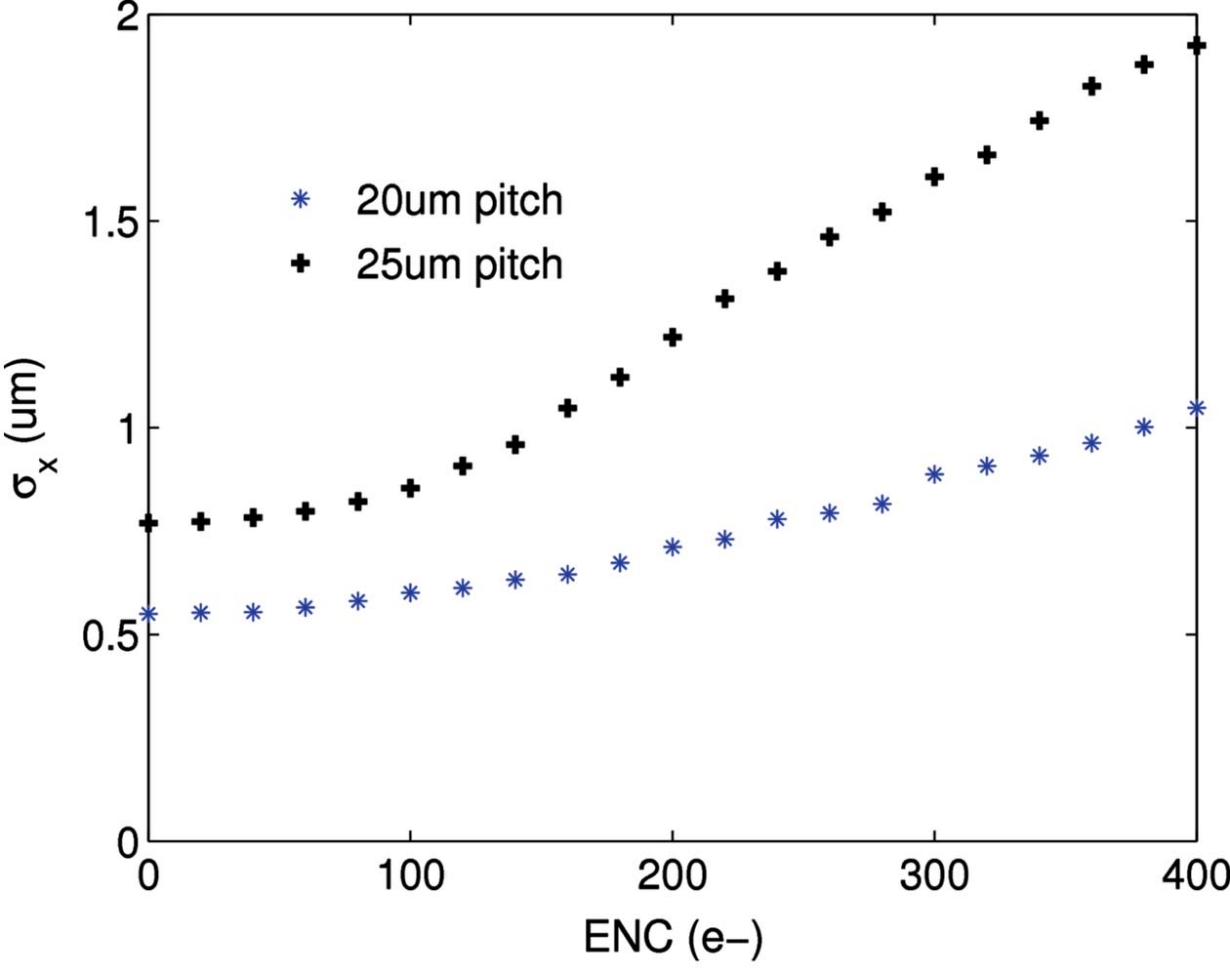
σ_*x*_ of the simulated PSF as a function of ENC for the 20 and 25 µm-pitch strips with a coupling of *K* = 0.06 at 15 keV. As expected, the spatial resolution degrades with noise.

Changing interstrip capacitance or noise leads to a significantly different η distribution (see Figs. 3 ▶ and 4 ▶) which degrades the performance of the η algorithm. In the experiment, significant variation is observed in the noise and gain levels between strips of the same pitch; therefore simulation suggests the η algorithm should be applied independently for every channel pair to optimize the spatial reconstruction. The 20 µm pitch shows greater charge sharing than the 25 µm pitch (see Fig. 5 ▶); therefore the reconstruction error for a flat-field illumination is significantly lower for the 20 µm pitch as seen in Fig. 6 ▶ (top) for the same noise level. Fig. 6 ▶ (bottom) also shows the reconstruction error for photons injected at the strip centre and close to the strip boundary. As expected, the reconstruction error is much larger at the strip centre owing to the much reduced charge sharing. A Gaussian fit to the data results in a reconstruction error σ_*x*_ of 1.38 ± 0.02 µm at the strip centre and 0.40 ± 0.03 µm close to the strip boundary (note that FWHM = 2.35σ_*x*_). This effect can also be seen in the experimental data (see §5 and Fig. 9). As a result of the simulation, Fig. 7 ▶ shows the resolution as a function of ENC for 20 and 25 µm pitch.

## 5. Experimental set-up

**Figure 8:**
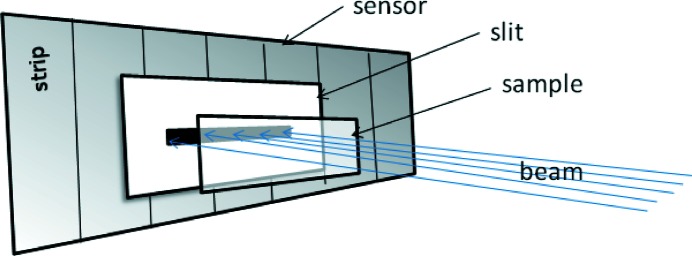
Simplified diagram (not to scale) indicating the positions of the strip sensor, the 2 µm slit, the sample and the direction of the incoming parallel beam for all microstructure scans. The sample is scanned vertically or horizontally depending on the measurement.

The following analyses are based on the 20 µm-pitch sensor for 15 keV X-rays as this provides the best results. This presumption is supported by previous experiments as well as simulation. A simplified diagram of the experimental set-up is shown in Fig. 8 ▶. The sample is mounted on a submicrometre-precision linear stage, allowing either horizontal or vertical motion as well as rotation about the beam axis. The stages have a linear repeatability of 0.4 µm. Upstream tungsten slits permit shaping of the beam impinging on the set-up.

The integration time is selected such that the rate of the impinging photons per channel is one every few frames, ensuring isolated hits in each frame, *i.e.* at least one unoccupied strip on either side of the hit position. The difference between the pre- and post-integration values is found followed by a pedestal subtraction and gain correction. Then the η value is calculated for each hit from which the position of the hit is reconstructed using equation (2). Before any measurements are performed, a flat-field exposure is used to ensure uniform illumination of all channels from which the relation between η and position is calculated independently for every strip pair to account for variability between strips. Simulation (see Fig. 7 ▶) indicates that the spread in noise values for 20 µm-pitch strips, 334 ± 8 e^−^, amounts to a spatial resolution variation of approximately 10% across the sensor. The strips chosen in the analysis are representative of the average noise values and can therefore be said to yield the average spatial resolution for the sensor.

### 5.1. Spatial resolution measurement

**Figure 9:**
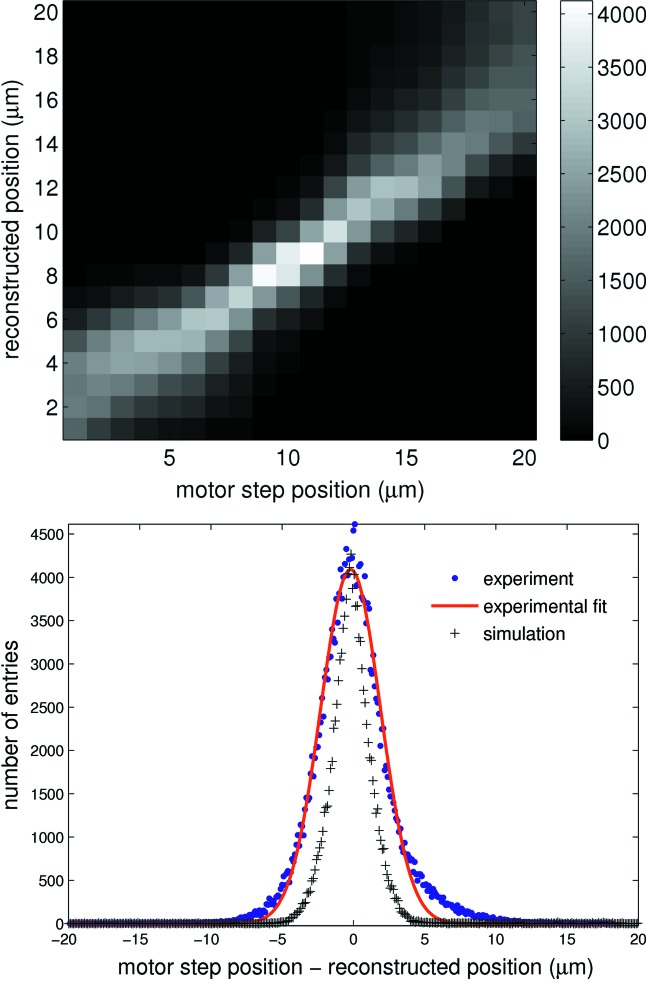
Top: motor position *versus* reconstructed position giving an indication of the reconstruction error. A 20 µm segment spanning two strips, with 0 µm and 20 µm corresponding to adjacent strip centres, is shown. Blurring of the line is a maximum at the strip centres. Bottom: quantifying the reconstruction error. This is approximately equivalent to the detector PSF for which the σ_*x*_ of the experimental profile is measured to be 1.8 µm. The simulated PSF for a 2 µm-wide square beam with ENC = 340 e^−^ and a coupling of *K* = 0.06 is also shown.

The spatial resolution of a system may be determined by measuring its response to a point source; this is termed the point-spread function (PSF), which is experimentally attained here and validated *via* simulation. A 2 µm tungsten slit is placed in front of the sensor parallel to the strips and scanned across several strips in 1 µm increments with 3 × 10^4^ frames acquired at each step. Comparing motor position to reconstructed position, as shown in Fig. 9 ▶, yields the error on the reconstructed position, which is a convolution of the PSF and experimental parameters such as the motor precision and tungsten slit width. A larger reconstruction error is observed at the strip centres [as predicted by simulations in Fig. 6 ▶ (bottom)] owing to noise having greater impact in this region of the η distribution.

The asymmetry observed in the PSF in Fig. 9 ▶ is due to an asymmetry in the scan region. From Fig. 9 ▶ the PSF is calculated to be approximately Gaussian with σ_*x*_ ≃ 1.8 ± 0.1 µm; this forms a conservative estimate of the resolution as the contribution from the beam width and motor resolution are not subtracted. The experimental PSF is compared with a simulation of a 2 µm square-profile beam scanned over several strips and is calculated *via* the same reconstruction procedure as outlined here with the exception of adjusting for gain variation and DC offset, as these are not present in the simulated data owing to the exclusion of all electronics. Simulation of a 2 µm-wide square beam scanned over adjacent strips is shown for comparison in Fig. 9 ▶; the resulting PSF is a Gaussian distribution with σ_*x*_ ≃ 1.3 ± 0.1 µm for an ENC of 340 e^−^. The difference between simulation and measurement stems from effects which are not taken into account in the simulation such as the beam divergence and the uncertainty in the alignment of the slit parallel to the strips. The uncertainty of the alignment is estimated to be 1°, with a beam height of 100 µm; this already causes an uncertainty of 1.7 µm. The resolution measurement is therefore currently limited by our ability to align the slit parallel to the strips. Fig. 7 ▶ demonstrates a lower limit for the reconstruction error of σ_*x*_ ≃ 0.8 µm for the 20 µm-pitch strip. With an optimized set-up, in particular in terms of slit size, a resolution of the order 1 µm should be achievable.

### 5.2. Periodic structures

**Figure 10:**
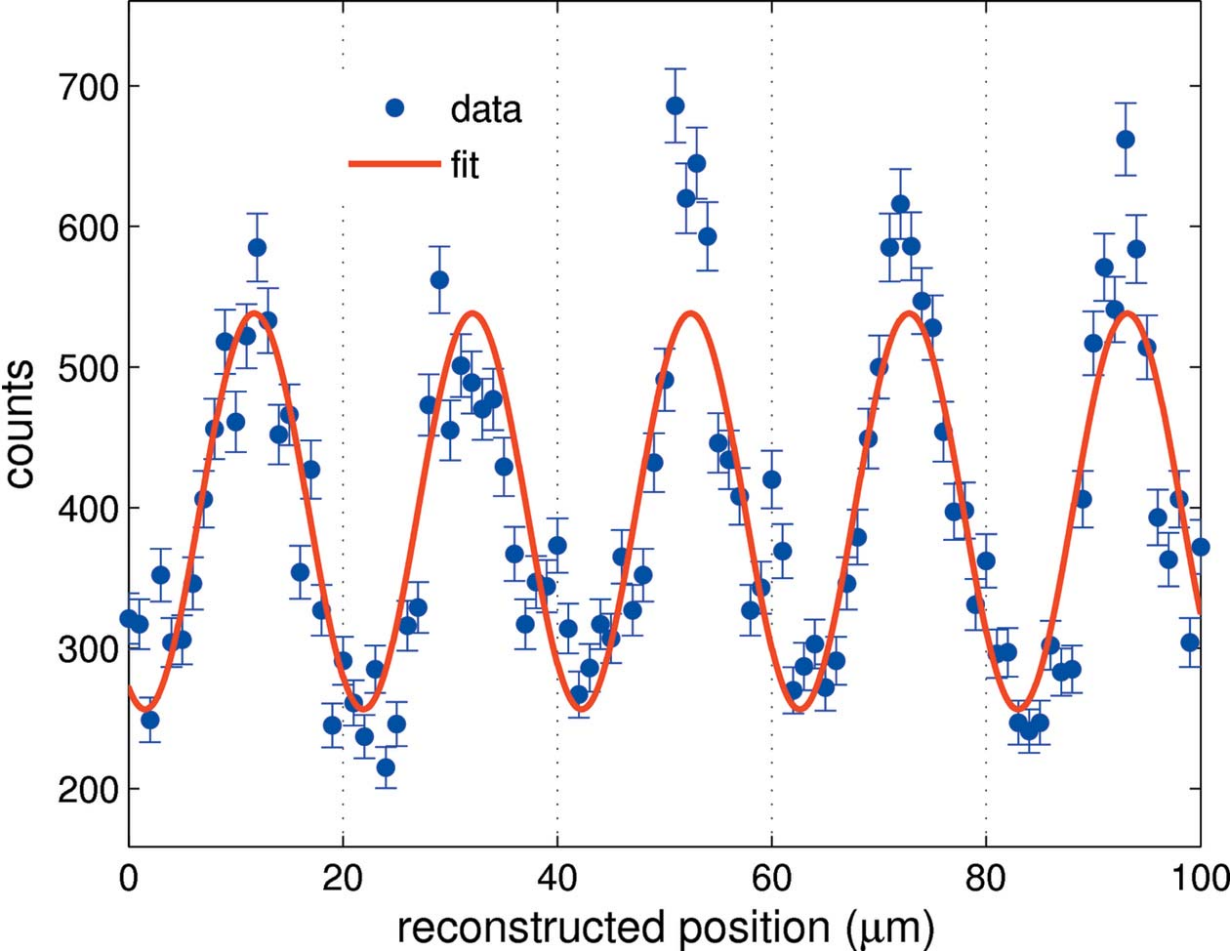
The 10 µm-wide ∼3 µm-thick Au lines on 300 µm Si show 52% contrast at 15 keV. The average FWHM measured is 10.4 µm. A sinusoidal fit is used to show the periodic nature of the structure.

The applicability of the reconstruction algorithm is tested with scans of various microstructures. The structures are formed *via* ∼3 µm-thick gold on 300 µm silicon and include a series of lines of varying thickness, where the pitch is equal to twice the strip width. The 2 µm slit is placed a few millimetres in front of the sensor, as shown in Fig. 8 ▶, whilst the gold line sample is kept statically in front of the slit with the sample lines orientated parallel to the strips. 2 × 10^5^ frames are acquired with an integration time of 1 µs. Even though the fabrication of narrower lines on the test sample is in principle possible (Gorelick *et al.*, 2010 ▶), line widths of 10 µm or greater were chosen for testing owing to their adequate quality and ease of alignment. As seen in Fig. 10 ▶, the measured contrast of the 10 µm lines is 52 ± 8%, from which the thickness of the gold layer is estimated to be 2.2 ± 0.2 µm, which is compatible with the sample character. The line width is measured to be 10.4 ± 0.9 µm and therefore demonstrates the possibility of reconstructing structures smaller than the strip pitch. The tungsten slit scan confirms that smaller structures down to sub-2 µm are resolvable, prompting a future attempt at producing and measuring the smaller pitch gold structures.

### 5.3. Complex structure test

**Figure 11:**
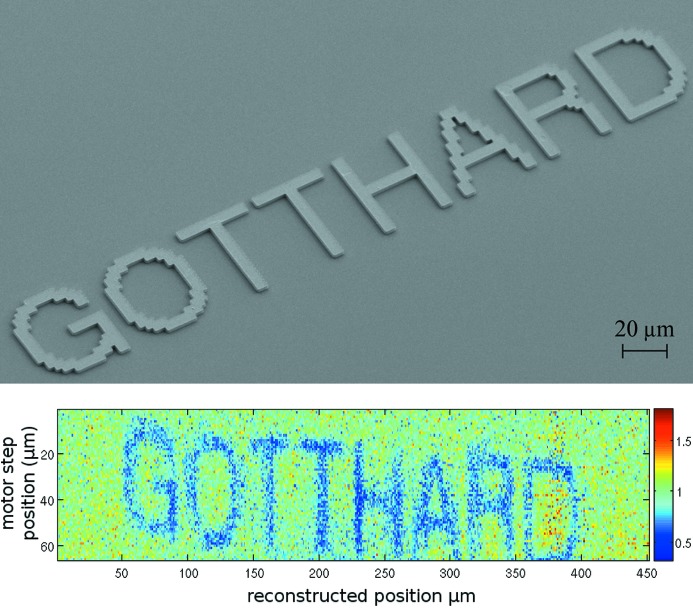
Top: scanning-electron-microscope image taken of 3 µm-thick Au letters of height 50 µm on 300 µm Si at 4.83 keV. Bottom: X-ray image reconstructed using the η algorithm. The angle and modulation in the reconstructed image are due to slight misalignment between the sample and strips and the shape of the collimating slit, respectively.

The experimental procedure is as described in the previous section (§5.2); however, the sample is now scanned vertically, and 2 × 10^4^ frames per 1 µm motor step are acquired. The charge interpolation algorithm is applied as for the tungsten slit scan. The reconstructed image seen in Fig. 11 ▶ is on an angle and contains a modulation owing to misalignment of the sample with respect to the strips and distortions in the slit, respectively.

The line width of the letters is ∼7 µm as seen in the scanning-electron-microscope image in Fig. 11 ▶. Averaged cross sections of the vertical components of the letters H and T are used to determine the FWHM of the lines to be 8.6 ± 1.3 µm.

## 6. Conclusions and outlook

It has been demonstrated that the charge integrating chip GOTTHARD in combination with a 20 µm-pitch strip sensor is capable of resolving sub-2 µm structures using a non-linear charge interpolation approach. The simulation of the resolution shows that the measurement is limited by the experimental set-up and not the detector. The reconstruction of 50 µm-high letters demonstrates the high-resolution imaging capability of the charge integration approach over single-photon-counting systems at low count rates. If the noise is reduced to 100 e^−^, as is the case for a pixel detector, then from Fig. 7 ▶ it is predicted that submicrometre spatial resolution may be achieved.

## Figures and Tables

**Figure 4:**
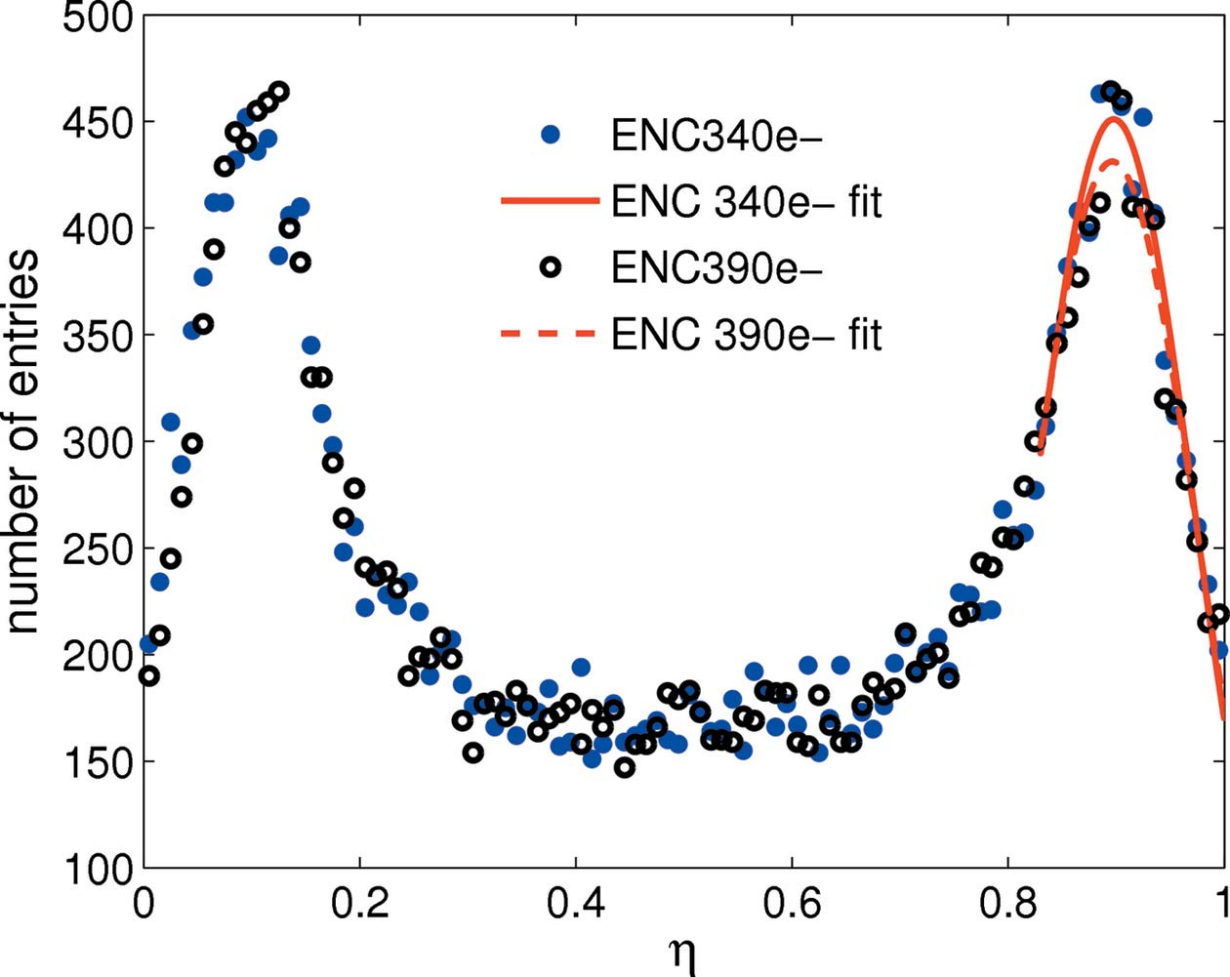
Simulated η distribution for ENC = 340 and 390 e^−^ which correspond to the minimum and maximum noise values measured for the 20 µm-pitch strips. The Gaussian fits show a broadening of the lateral peaks with increased noise.
